# Two treatments, one disease: childhood malaria management in Tanga, Tanzania

**DOI:** 10.1186/1475-2875-8-240

**Published:** 2009-10-27

**Authors:** Deshka Foster, Stacie Vilendrer

**Affiliations:** 1 Program in Human Biology, Stanford University, Stanford, CA, USA

## Abstract

**Background:**

In the Tanga District of coastal Tanzania, malaria is one of the primary causes of mortality for children under the age of five. While some children are treated with malaria medications in biomedical facilities, as the World Health Organization recommends, others receive home-care or treatment from traditional healers. Recognition of malaria is difficult because symptoms can range from fever with uncomplicated malaria to convulsions with severe malaria. This study explores why caregivers in the Tanga District of Tanzania pursue particular courses of action to deal with malaria in their children.

**Methods:**

Qualitative data were collected through interviews with three samples: female caregivers of children under five (N = 61), medical practitioners (N = 28), and traditional healers (N = 18) in urban, peri-urban, and rural areas. The female caregiver sample is intentionally stratified to reflect the greater population of the Tanga District in level of education, marital status, gender of household head, religion, and tribal group affiliation. Qualitative data were counted, coded and analysed using NVivo7 software.

**Results:**

Results indicate that a variety of factors influence treatment choice, including socio-cultural beliefs about malaria symptoms, associations with spiritual affliction requiring traditional healing, knowledge of malaria, and fear of certain anti-malaria treatment procedures. Most notably, some caregivers identified convulsions as a spiritual condition, unrelated to malaria. While nearly all caregivers reported attending biomedical facilities to treat children with fever (N = 60/61), many caregivers stated that convulsions are best treated by traditional healers (N = 26/61). Qualitative interviews with medical practitioners and traditional healers confirmed this belief.

**Conclusion:**

Results offer insight into current trends in malaria management and have implications in healthcare policy, educational campaigns, and the importance of integrating traditional and biomedical approaches.

## Background

Malaria remains one of the greatest public health challenges of our time. In the United Republic of Tanzania, malaria is a leading cause of mortality for children under five [1]. While some children are treated with malaria medications from biomedical facilities as the World Health Organization recommends, others receive treatment at home or from traditional healers [2]. Investigating social and cultural issues regarding malaria treatment is integral in the development of effective public health responses to the disease [3].

The Tanzanian government developed the Tanzania National Malaria Medium Term Strategic Plan for 2002 to 2007, which aimed to reduce malaria by 25 percent by 2007 and 50 percent by 2010 [4]. This plan employs a multi-faceted approach, incorporating free or low-cost artemisinin-based combination therapy (ACT) in public hospitals, insecticide-treated bed nets (ITNs), indoor residual spraying (IRS), intermittent preventive treatment in pregnancy (IPTp), training nurses in malaria case-management, and support for community education and ongoing research. However, malaria rates continue to rise in some regions of Tanzania; the plan did not meet its 2007 first target goal and will likely not meet its second target goal for 2010. In addition, recent evidence indicates that only 34 percent of malaria cases in children under the age of five are appropriately managed in biomedical facilities, leaving the vast majority of cases without adequate care [5].

The Tanzania National Malaria Control Programme works with donor agencies to coordinate efforts in Tanzania's malaria programme. The country receives significant funding for these activities through the US Government's President's Malaria Initiative (PMI), the Global Fund to Fight AIDS, Tuberculosis and Malaria (GFATM), the World Bank, and other public and private organizations. The "Under Five Catch-Up Campaign" launched in December 2008 is designed to distribute free bed nets to all children up to five years of age. The Government of Tanzania has additional plans to distribute 14.6 million long-lasting insecticide-treated bed nets (LLTNs) with the target of achieving 2.5 nets per household in 2010. Tanzania also recently implemented programs to promote behaviour change. In October 2007, PMI launched the "Communications and Malaria Initiative in Tanzania" (COMMIT) to address household behaviours such as proper use of ITNs, ACTs, and IPTp. The associated mass media campaign aims to reach 80 percent of the population nationwide [6].

A review of the relevant literature reflects a shift away from implicating the caregiver in cases of sub-optimal childhood malaria treatment (i.e. waiting too long to bring the child to a hospital), to recognizing the social and economic constraints of the environment [7]. More recent studies suggest specifically that caregivers choose the treatment they perceive to be most effective within the constraints of their environment [7,8]. According to Comoro *et al *[9], increased knowledge of caregivers' "local understanding, perceptions, and practices" regarding the treatment of childhood malaria is crucial to improving malaria management.

Several factors have been cited as influencing treatment-seeking behaviour in the case of childhood malaria. These include socio-cultural beliefs [10], cost [11,12], distance to a medical facility [12,13], and gender dynamics within the household [8,13,14]. The specific symptoms of malaria have also been shown to influence a caregiver's treatment of her child's illness [8,9,13,15]. In general, caregivers seem to associate fever with malaria [16] and consequently attend biomedical facilities [17]. This study investigates why caregivers in the Tanga District pursue particular courses of malaria treatment for their children.

Studying malaria treatment at the local level in Africa would be incomplete without considering the role of traditional healers and traditional medicines, as traditional healers represent the first line of care for over 70 percent of the population in Tanzania [18,19]. Currently, malaria is diagnosed and treated by traditional healers in a variety of ways including biomedical, traditional, or combinations of both, depending on the symptoms [6,20]. The literature has shown repeatedly that traditional healers are consulted most often when the cause of illness is believed to be spiritual or demonic [12,17]. A traditional healer may prescribe an array of treatments for a child with malaria including herbal remedies, such as prepared plants or roots, or spiritual remedies, such as exorcism [11].

The Tanga District is known both for high rates of malaria and its vast network of traditional healers [11,19]. With a population of over 240,000, the district is subdivided into 24 wards of varying size and population, and contains three large district hospitals and many smaller clinics [21].

## Methods

### Data collection

Data for this exploratory study were collected June-August, 2007 in the form of one-time, semi-structured interviews with three sample groups: female caregivers of children under five (N = 61), medical practitioners (N = 28), and traditional healers (N = 18) from urban, peri-urban, and rural wards within the Tanga District (Table 1).

**Table 1:** Sample of caregivers and traditional healers by location

Location	Female Caregivers, N	Traditional Healers, N
**Urban**	21	6

**Peri-Urban**	18	6

**Rural**	22	6

**Total**	61	18

All interviews were conducted by the primary investigators in Swahili and translated to English with the help of native Swahili speakers. Interviews were recorded and transcribed for analysis.

The female caregiver sample was purposefully stratified to reflect the overall population of the Tanga District in terms of level of education, marital status, gender of household head, religion, and tribal group affiliation.

Medical practitioners, including physicians, nurses, clinical officers, laboratory technicians, and pharmacists were interviewed from a variety of public, private, urban, and rural healthcare facilities in the Tanga District (Table 2).

**Table 2:** Medical practitioner by location, type of workplace, and professional title

Location	Public	Private	Pharmacy	Total
**Urban**	1 Medical Doctor	2 Medical Doctors	2 Pharmacists	17
	1 Clinical Officer	1 Assistant Medical		
	3 Nurses	Officer		
	2 Medical Assistants	1 Clinical Officer		
	1 Regional Director of	3 Nurses		
	Malaria Program			

**Peri-urban**	1 Nurse	N/A	N/A	2
	1 Clinical Officer			

**Rural**	3 Clinical Officers	1 Clinical Officer	N/A	9
	4 Nurses			
	1 Maternal and Child			
	Health Aid			

**Sample Total**	18	8	2	28

The Tanga AIDS Working Group (TAWG), a non-governmental organization based in the city of Tanga, provided a database of traditional healers within the Tanga District, from which a sample of healers was randomly selected for participation in this study. For all sample groups, the primary investigators were introduced to research participants by local informants, affording a greater level of trust than might be achieved without a facilitated introduction.

### Data analysis

Prior to field research, the primary investigators developed a conceptual framework targeting the central research question. In the framework's most simple form, several factors were identified to influence treatment-seeking behaviour - the decision as to when and where a child should be taken for treatment. As advocated by Miles and Huberman [22], this framework was used to guide the research analysis, in which the primary investigators used interview data to identify and corroborate newly emergent themes [22]. Qualitative interview data were counted, coded and analysed using qualitative research software (NVivo7). A multistage coding strategy was employed following the "grounded" approach advocated by Glaser [23], coding for words and ideas as they relate to the research questions. Three basic levels of codes were used: descriptive, interpretive, and pattern/thematic [23].

### Ethical review

The study underwent ethical review and received approval from Stanford University's Internal Review Board (IRB) Research Compliance Office and the Tanzania Commission for Science and Technology in Dar es Salaam, Tanzania. The primary investigators also obtained letters of introduction and support from the Ministry of Health Tanga Regional Office, the Chief Clinical Officer of Bombo Regional Hospital in the Tanga District, and the TAWG.

## Results

Of the factors evaluated, those identified as influential in caregivers' treatment decisions for childhood malaria included socio-cultural beliefs surrounding malaria symptoms, associations with spiritual affliction requiring traditional healing, knowledge of malaria, and fear of biomedical treatment procedures. Of these, the most significant is the existence of separate treatment trends for uncomplicated and severe malaria, which seem to be motivated by beliefs associated with the specific symptoms a child develops.

### Two profiles of malaria

Malaria has remained a public health challenge in part because the disease is often difficult to diagnose. Symptoms range from fever, headache, malaise, diarrhoea and vomiting with uncomplicated malaria to convulsions with severe or complicated malaria [2]. In children, malaria can quickly develop into severe malaria without prompt treatment, putting children at high risk for permanent brain damage or death.

Caregivers showed a high level of knowledge regarding the prevention, diagnosis and treatment of uncomplicated malaria, but had differing views about the cause and symptoms of severe malaria. The vast majority of caregivers identified mosquitoes as the cause of malaria and cited several symptoms of uncomplicated malaria; many could even mention a specific treatment. However, when it came to severe malaria, caregiver knowledge was highly variable. While about half of the caregiver sample (N = 37/61) identified convulsions as a symptom of severe malaria in children, the others reported that convulsions signify a separate disease, distinct from malaria, with its origins in the spiritual world.

In order to assess the influence of malaria symptoms on treatment-seeking behavior, it is necessary to develop a vocabulary of specific malaria terms in Swahili [8]. Interviews with research participants revealed a set of Swahili words used to describe convulsions and their associated illness. Female caregivers were initially reluctant to mention terms relating to severe malaria, likely because of their spiritual connotations. However, when prompted, the two terms most frequently employed were *dege dege *and *mchango*, both of which have a variety of definitions and strong associations with spiritual affliction. Some female caregivers offered explanations of these two terms, claiming that the specific presentation of *dege dege *or *mchango *depends on the child, "*for one baby, it is turning the eyes upside down. For another one it is high fever*" (Caregiver #10). Other Swahili words mentioned in association with convulsions included *uchawi, upepo*, and *zongo*. The definition of each of these terms and their level of association with malaria varied significantly between each individual caregiver.

Similar to the female caregiver sample, the majority of medical practitioners defined the term *dege dege *as convulsions (N = 25/28), 46 percent of whom connected *dege dege *specifically with malaria convulsions (N = 13/28). Two-thirds of traditional healers associated *dege dege *with convulsions (N = 12/18), and only 33 percent suggested the likely cause to be malaria (N = 6/18).

Responses regarding the distinction between *dege dege *and *mchango *were highly variable. Seventy-one percent of medical practitioners reported that *dege dege *and *mchango *are exactly the same (N = 21/28), whereas only 56 percent of traditional healers (N = 10/18) equated the terms. One third of traditional healers reported that *dege dege *and *mchango *are unrelated diseases (N = 6/18). In all three of the samples groups, association between convulsions and *dege dege *and/or *mchango *was relatively high, suggesting that *dege dege *and *mchango *have a level of common identification in convulsions, independent of the level of spiritual belief associated with the term.

Close to half of the traditional healers reinforced this non-biomedical understanding of convulsions. Of the 18 traditional healers in the sample, eight said that they believed the same symptoms could have both biomedical and spiritual causes:

*I know that fever is caused by a virus. But when I think it is malaria, I tell the people to go to the hospital... Other times when it is not caused by malaria, it can be caused by demons. When it is demons, I can treat them...I have some drugs that I am using to treat those demons. I call them to talk with them*. (Traditional healer #4)

All of the medical practitioners interviewed; however, identified convulsions as the most common manifestation of severe malaria and stated that such symptoms must be treated biomedically. They almost always recommended injections of quinine as the optimal treatment.

Qualitative evidence from caregivers suggests that an individual's level of knowledge of malaria influences whether convulsions are considered a sign of severe malaria or a spiritual illness: *"Some parents, they just follow beliefs. They are not educated, so they don't know. They go to see the child vomiting, you know, and think it is demon stuff" *(Caregiver #7). Multiple medical practitioners also cited a lack of education as a reason that caregivers chose to go to a traditional healer over a biomedical facility.

### Treatment-seeking behaviour for uncomplicated malaria

The majority of female caregivers reported that they sought treatment at a biomedical facility, such as a hospital or clinic, the last time they suspected that their child had malaria (N = 57/61). Three went to a traditional healer either before or after going to a biomedical facility, and only one sought treatment entirely outside of a biomedical facility - at her local pharmacy.

Several caregivers mentioned wanting a malaria test as the primarily reason for going to a clinic or hospital. They seemed to directly associate a malaria test with good treatment: *"I was satisfied because I got treatment and testing" *(Caregiver #82). Both medical practitioners and traditional healers generally corroborated this trend in treatment-seeking behaviour, identifying that caregivers usually take their children to biomedical facilities for uncomplicated malaria.

### Severe malaria management

When caregivers were questioned about how to manage symptoms relating to severe malaria, such as convulsions, an alternate trend emerged. Nearly a quarter stated that a caregiver should seek the advice of a traditional healer if a child has convulsions (N = 15/61). Another 18 percent said that it does not matter whether a child goes to a biomedical facility or a traditional healer (N = 11/61), and 28 percent said that they were not sure how best to manage a convulsing child (N = 17/61). Unlike with uncomplicated malaria, caregivers were less certain of the appropriate treatment for convulsions.

Of the 43 percent (N = 26/61) that mentioned traditional healing as an appropriate treatment for a child with convulsions, almost all reported that convulsions are the sign of spiritual diseases most commonly called *dege dege *and *mchango*. Several caregivers also mentioned using traditional treatments themselves for *dege dege *and *mchango *rather than attending a healer:

*Since I gave birth to my baby, I have never taken my baby to the traditional healer. But there are some diseases like mchango where, when it comes, I take some herbs, I go myself and take some herbs and apply to the body of the baby and it recovers from disease*. (Caregiver #20)

Within traditional treatments described for *dege dege *and *mchango *there is significant variability. Remedies described by caregivers ranged from herbs that can be boiled and consumed as a tea or showered in, to the use of elephant dung, indigenous hens, and spiritual and religious rituals:

*For mchango, traditional healers take garlic peels and they mix with elephant waste and they heat the garlic peels. Then they take a piece of kanga [cloth] and cover the baby so that the smoke can spread it in the body of the baby. If you use the first treatment and it fails, there are other traditional treatments that you can use like the leaves of the plant over there [she points to a bush across the yard]. We scratch the leaves and mix with water and then we wash the baby in the water of the leaves from the plant from over there*. (Caregiver #17)

Several of these caregivers not only said that traditional healing is the way to cure convulsions, but also mentioned that anti-malarial injections (such as quinine) from biomedical facilities, used to treat children with *dege dege *or *mchango*, are dangerous. Traditional healers also brought up this belief, often using it to justify traditional medicine:

*For convulsions most people take their children to a traditional healer. The reason behind not going to the hospital is that when they go to the hospital, they tend to inject the child. When they get the injection, they will be paralyzed. The traditional healer will be able to relieve the convulsions*. (Traditional healer #11)

In this case, beliefs about malaria symptoms are strengthened by a fear of injections, persuading caregivers to seek traditional treatment rather than attending a biomedical facility.

### Traditional healers in the biomedical community

Despite the aforementioned suspicions of specific biomedical treatments, traditional healers reported a notable commitment to working alongside the biomedical community. Thirteen of the 18 traditional healers interviewed reported sending patients to biomedical facilities for malaria testing, even if they ultimately intended to treat them with traditional remedies:

*Many come here first. I can treat the symptoms, but I send them to the hospital to test first and then I treat them. It is most important that people go and get tested early enough*. (Traditional healer #15)

These findings support McMillen's findings [19], which conclude that traditional healers are generally cooperative within the biomedical community. Specifically, traditional healers seem to understand that their unique skills are not as demanded as they once were and are adapting to the new environment by interacting with the biomedical community [14].

Traditional healers tend to make all or part of their living from healing, which can influence how they are viewed in the community. In this study, of the 18 traditional healers interviewed, 55 percent reported they work full-time as a healer (N = 10), while 38 percent have other jobs (N = 7), and one had retired from an earlier profession to do healing full time. Some caregivers were suspicious of the fact that traditional healers make their livelihood from healing:

*For me, I have not gone to a traditional healer. Many of them are cheating. If you go to the healers, they will just be cheating and say, 'Oh this is zongo' because they need money*. (Caregiver #52).

Several caregivers reported avoiding traditional healers for such reasons, despite the desire to cooperate with the biomedical community noted by the traditional healers.

## Discussion

### Malaria as two diseases

The understanding of convulsions as a separate illness from malaria is highly disconcerting from a public health perspective. In this study, an alarming eight percent of the caregivers interviewed reported having had a child die from malaria. As the evidence suggests, if caregivers do not understand the full range of symptoms associated with malaria, they may not know to find the appropriate treatment. Further, some traditional healers may not recognize severe malaria, which can further delay and potentially prevent the administration of biomedical treatments.

Why might some caregivers believe that convulsions are caused by malaria while others believe they signify a separate illness? Qualitative evidence from both caregivers and medical practitioners suggests that an individual's knowledge of malaria influences whether convulsions are considered a sign of severe malaria or spiritual illness. Of the caregivers that mentioned traditional healing as the most appropriate treatment for a child with convulsions, almost all reported that convulsions are the sign of a spiritual disease, usually *dege dege *and/or *mchango*. Their responses suggest the belief that convulsions signify a disease that is distinct from malaria, which requires traditional healing. A lack of education about malaria and its full range of symptoms may be a primary reason for caregivers to choose traditional healing over a biomedical facility.

In this study, caregivers reported receiving malaria education through public health campaigns, medical professionals, schools, and other members of the community. The evidence suggests that prior public health malaria awareness efforts have influenced treatment-seeking behaviour for malaria. Caregivers seem to recognize the symptoms and danger of uncomplicated malaria and nearly always chose biomedical care. However, these efforts do not seem to have fully informed caregivers and traditional healers that convulsions are a frequent symptom of severe malaria, which requires urgent, biomedical treatment. Information on convulsions should be incorporated into malaria campaigns, particularly mass media efforts designed to promote behaviour change.

While enhancing education about the symptoms of severe malaria may mitigate the problem, the current healthcare conditions call for a multi-faceted approach. Malaria deaths often occur because children do not receive appropriate malaria medications in time to save their lives. Unfortunately, medication for severe malaria, including injections of benzodiazepam (Valium^®^) and quinine, are often only available at large clinics and hospitals, and the referral process from a rural dispensary to an urban hospital can delay treatment. One possible solution may be to equip rural dispensaries with medications for severe malaria and train staff to manage severe malaria symptoms, such as convulsions. However, current limitations in economic and human resources in Tanzania challenge the feasibility of such a policy change.

### The role of traditional healers

Findings show that the current role of traditional healers in malaria management is complex. Traditional healers and herbal treatments were found to play an even smaller role than anticipated for uncomplicated malaria. Although traditional healers are located within the community and are generally available to treat children for a nominal fee, caregivers prefer to go to biomedical facilities for uncomplicated malaria. Traditional healers play a more significant role in cases of severe malaria--when people believe biomedicine is less effective. A few caregivers reported using both traditional healers and biomedical facilities for the same episode of illness, going from one to the other in sequence.

Some healers use biomedicine in conjunction with their own practice, as in the case of malaria testing. As caregivers were found to associate malaria testing with good treatment, traditional healers may be adapting to their clientele's changing demands by sending caregivers to biomedical facilities for a malaria test. Given this trend, it may be both feasible and necessary for traditional healers to work more closely with the biomedical community. Traditional healers are in a position to dispense not only treatment but also education to their immediate communities. A concerted educational campaign targeting the prevention and treatment of malaria, directed towards traditional healers, could impact a significant number of caregivers. As traditional healers adapt to new roles in a changing healthcare structure, they may become an important resource for malaria information. Such collaboration has been achieved for HIV/AIDS treatment and prevention by the Tanga AIDS Working Group, a non-profit organization that has educated hundreds of traditional healers on the biology of the disease with successful results.

## Conclusion

The results offer insight into current trends in malaria management and have implications in healthcare policy and educational campaigns. Caregivers in the Tanga District understand malaria transmission, prevention, and treatment. They overwhelmingly choose treatment from a biomedical facility when their children have malaria. However, a significant portion does not associate convulsions with severe malaria and instead believe that such symptoms are due to a separate illness with its origins in the spirit world.

These findings suggest limitations of the current malaria management and healthcare policy. Health education campaigns may be able to effectively reduce the severe malaria death toll by focusing on the symptoms of severe malaria. This could clarify the causes of convulsions, dispel associations with non-biomedical illnesses, and emphasize that severe malaria should be treated at biomedical facilities.

## Competing interests

The authors declare that they have no competing interests.

## Authors' contributions

DF and SV conceived of the study together, equally participated in design of the study, conducted the field research together, and co-authored this research project. Both authors read and approved the final manuscript.
